# Effect of 10 Minutes of Prewarming and Prewarmed Intravenous Fluid Administration on the Core Temperature of Patients Undergoing Transurethral Surgery under General Anesthesia

**DOI:** 10.7150/ijms.88943

**Published:** 2024-01-01

**Authors:** Joonho Cho, Jin-min Lee, Kye-Min Kim, Jun Heum Yon, Hye Sun Lee, In-Jung Jun

**Affiliations:** 1 Department of Anesthesiology and Pain Medicine, Inje University Sanggye Paik Hospital, Seoul, Korea.; 2 Biostatistics Collaboration Unit, Yonsei University College of Medicine, Seoul, Korea.

**Keywords:** Anesthesia, general, Hypothermia, Perioperative care, Transurethral resection of bladder, Transurethral resection of prostate

## Abstract

**Background:** Patients undergoing transurethral urologic procedures using bladder irrigation are at increased risk of perioperative hypothermia. Thirty minutes of prewarming prevents perioperative hypothermia. However, its routine application is impractical. We evaluated the effect of 10 minutes of prewarming combined with the intraoperative administration of warmed intravenous fluid on patients' core temperature.

**Methods:** Fifty patients undergoing transurethral bladder or prostate resection under general anesthesia were included in this study and were randomly allocated to either the control group or the prewarming group. Patients in the prewarming group were warmed for 10 minutes before anesthesia induction with a forced-air warming device and received warmed intravenous fluid during operations. The patients in control group did not receive preoperative forced-air warming and were administered room-temperature fluid. Participants' core body temperature was measured on arrival at the preoperative holding area (T_0_), on entering the operating room, immediately after anesthesia induction, and in 10-minute intervals from then on until the end of the operation (T_end_), on entering PACU, and in 10-minute intervals during the postanesthesia care unit stay. The groups' incidence of intraoperative hypothermia, change in core temperature (T_0_ - T_end_), and postoperative thermal comfort were compared.

**Results:** The incidence of hypothermia was 64% and 29% in the control group and prewarming group, respectively (P = 0.015). Change in core temperature was 0.93 ± 0.3 °C and 0.55 ± 0.4 °C in the control group and prewarming group, respectively (P = 0.0001). Thermal comfort was better in the prewarming group (P = 0.004).

**Conclusions:** Ten minutes of prewarming combined with warmed intravenous fluid significantly decreased the incidence of intraoperative hypothermia and resulted in better thermal comfort in patients undergoing transurethral urologic surgery under general anesthesia.

## Introduction

Inadvertent perioperative hypothermia, defined as a core temperature < 36.0 °C, is associated with various adverse outcomes, such as morbid cardiac events, delayed postanesthetic recovery, wound infection, and prolonged hospital stays 1-3. During general anesthesia, the body's ability to produce heat is reduced by a lowered threshold for coldness 4. Moreover, core-to-peripheral thermal redistribution leads to a rapid drop of the core body temperature within initial first hour of general anesthesia 5. Urologic procedures, such as transurethral resection of the bladder (TURB) or the prostate (TURP), require bladder irrigation with a massive amount of fluid, which makes the patient even more prone to hypothermia 6. A majority of patients undergoing TURB and TURP are elderly and the lithotomy position used in these procedures makes whole-body air warming impossible. All of these factors reflect the need for an efficient way to maintain normothermia.

Of the various ways to avoid perioperative hypothermia, prewarming, which is the warming of the body's surface before anesthesia induction, is effective and safe 7, 8. National Institute for Health and Care Excellence guidelines suggest that 30 minutes of prewarming prevents perioperative hypothermia 9. However, 30 minutes or more of prewarming can be impractical for routine clinical practice because it delays surgery, increases congestion in the preanesthesia care unit. Recent studies on prewarming for 10 minutes give conflicting results in avoiding perioperative hypothermia 10-12.

In this study, we evaluated the efficacy of 10 minutes of prewarming combined with warmed intravenous fluid administration at avoiding perioperative hypothermia.

## Method

### Study design

This study was approved by the Institutional Review Board of Sanggye Paik Hospital (approval no. 2021-05-004). It was a prospective, randomized, single-blinded, controlled study designed in accordance with Consolidated Standards of Reporting Trials guidelines for randomized clinical trials. Written informed consent was obtained from all participants. This study was registered at clinicaltrials.gov prior to patient enrollment (registration no. NCT04991272). It was conducted between July 2021 and August 2022 at a single university hospital.

### Participants

The participants were adults undergoing TURB or TURP under general anesthesia. The exclusion criteria were patient refusal; a pre-induction body temperature outside of the normal range, namely > 37.5°C or < 36.0°C; having moderate to severe cardiopulmonary or renal disease; having thyroid disease; and suspicion or diagnosis of infection. The dropout criteria were severe perioperative hemodynamic instability, conversion to open surgery, and the anesthesiologist's discretion. Sample size was determined by estimating a core temperature difference of up to 0.5°C as the desired therapeutic effect because this is the difference related to hypothermia-induced complications 13. The sample size for each group was determined to be 23 (ɑ = 0.05, ß = 0.9) using G Power version 3.1.9.4 (Franz Faul, Universitat Kiel, Germany). Assuming a dropout rate of 5%, 50 participants were required.

### Randomization

The participants were randomly placed in a group using computer-generated randomization. Allocation was concealed in sequentially numbered, opaque, sealed envelopes. On the day of the operation prior to patient arrival, an investigator who was not involved in data collection opened the envelope containing the patient's group placement. Outcome assessors were blinded. During the operation and in the postanesthesia care unit (PACU), data was recorded by a nurse blinded to the patient's group. We decided to dropout the case when blinding was not kept.

### Protocol

The ambient temperatures of the preoperative holding area, the operating room, and the PACU were maintained at 21-23°C. The fluid used for bladder irrigation was normal saline kept at room temperature.

Patients in the prewarming group were covered with a cotton blanket over a WarmTouch full-body forced-air blanket (Covidien, LLC, Mansfield, MA, USA) that covered them from their neck to their feet and were prewarmed for 10 minutes using a Bairhugger forced-air warming device model 505 (Arizant Healthcare, Eden Prairie, MN, USA) set on high, which corresponded to 43°C. On entering the operating room, 8 ml/kg of warmed Plasma Solution A intravenous fluid (HK inno.N, Seoul, South Korea), kept in a warming cabinet set at 41°C for at least 8 hours, was administered. From then on, intravenous fluid was administered at a rate of 2 ml/kg/hr.

Participants in the control group did not receive prewarming. On entering the operating room, 8 ml/kg of Plasma Solution A intravenous fluid (HK inno.N, Seoul, South Korea), kept at room temperature, was administered. From then on, intravenous fluid was administered at a rate of 2 ml/kg/hr.

All participants were covered with a COVIDIEN WarmTouch Upper Body Blanket (Covidien, LLC, Mansfield, MA, USA) and a forced-air warming device was used on its medium setting, corresponding to 38°C, throughout the operation. If the participant's core temperature fell below 35.0°C, the forced-air warming device setting was changed to high, corresponding to 43°C, and if the core temperature rose above 37.0°C, the device was turned off. In the PACU, whole-body warming was applied if the participant requested it. Participants in the PACU who shivered were administered meperidine at the discretion of an anesthesiologist.

After entering the operating room, all patients were monitored with standard monitoring devices, such as three-lead electrocardiography, pulse oximetry, and noninvasive blood pressure devices. General anesthesia was induced using balanced anesthesia. Loss of consciousness and neuromuscular blockade were achieved by administering intravenous 1-2 mg/kg of propofol and 0.6-0.8 mg/kg of rocuronium. After endotracheal intubation, anesthesia was maintained with sevoflurane and remifentanil using target-controlled infusion.

### Data Collection

Intraoperative hypothermia was defined as a temperature measured in the esophagus or tympanic membrane <36.0°C at the end of the surgery. The severity of hypothermia was classified as mild (35.0-35.9°C), moderate (34.0-34.9°C), or severe (< 34°C) 14.

Participants' core body temperature was measured on arrival at the preoperative holding area (T_0_), on entering the operating room, immediately after anesthesia induction, and in 10-minute intervals from then on until the end of the operation (T_end_), on entering PACU, and in 10-minute intervals during the PACU stay. The temperatures measured during anesthesia were obtained using an Esophageal Stethoscope (Erae SI Co., Ltd., Seoul, South Korea). The sensor was positioned 28-32 cm from the upper incisors. The temperatures measured while the patient was awake were obtained using a Thermoscan IRT tympanic thermometer (Braun, Kronberg, Germany). In this case, the highest of three sequential measurements was recorded.

The change in perioperative core temperature was calculated as T_0_ - T_end_ and the core temperature drop rate was defined as T_0_ - T_end_ / [anesthesia time]. Incidence of shivering was checked. It was measured on entering the operating room, on entering the PACU, and in 10-minute intervals from then on. Thermal comfort was measured on entering the operating room and right before the departure from the PACU using a 10-point scale in which 0 = extremely cold, 5 = thermally neutral, and 10 = extremely hot. A thermal comfort score of 5 meant that the patient was the most thermally comfortable. Scores lower and higher than 5 indicated that the patient felt cold and hot, respectively.

The primary outcome was the incidence of intraoperative hypothermia. Secondary outcomes were change in perioperative core temperature, postoperative shivering, and postoperative thermal comfort.

### Statistical Analysis

Statistical analysis was performed using SPSS version 22.0 (SPSS Inc., Armonk, NY, USA) or SAS version 9.4 (SAS Institute Inc., Cary, NC, USA). The data was analyzed to determine if it was normally distributed using Kolmogorov-Smirnov tests. The groups' demographic and perioperative data was compared using Student's t-tests for continuous variables and the Chi-squared test for categorical variables. Mann-Whitney U tests or Fisher's exact tests were used for nonparametric data analyses. Core temperature changes were compared between the two groups using a linear mixed model in SAS version 9.4 (SAS Institute Inc., Cary, NC, USA). P-values < 0.05 were considered statistically significant in all analyses.

## Results

**Figure 1:**
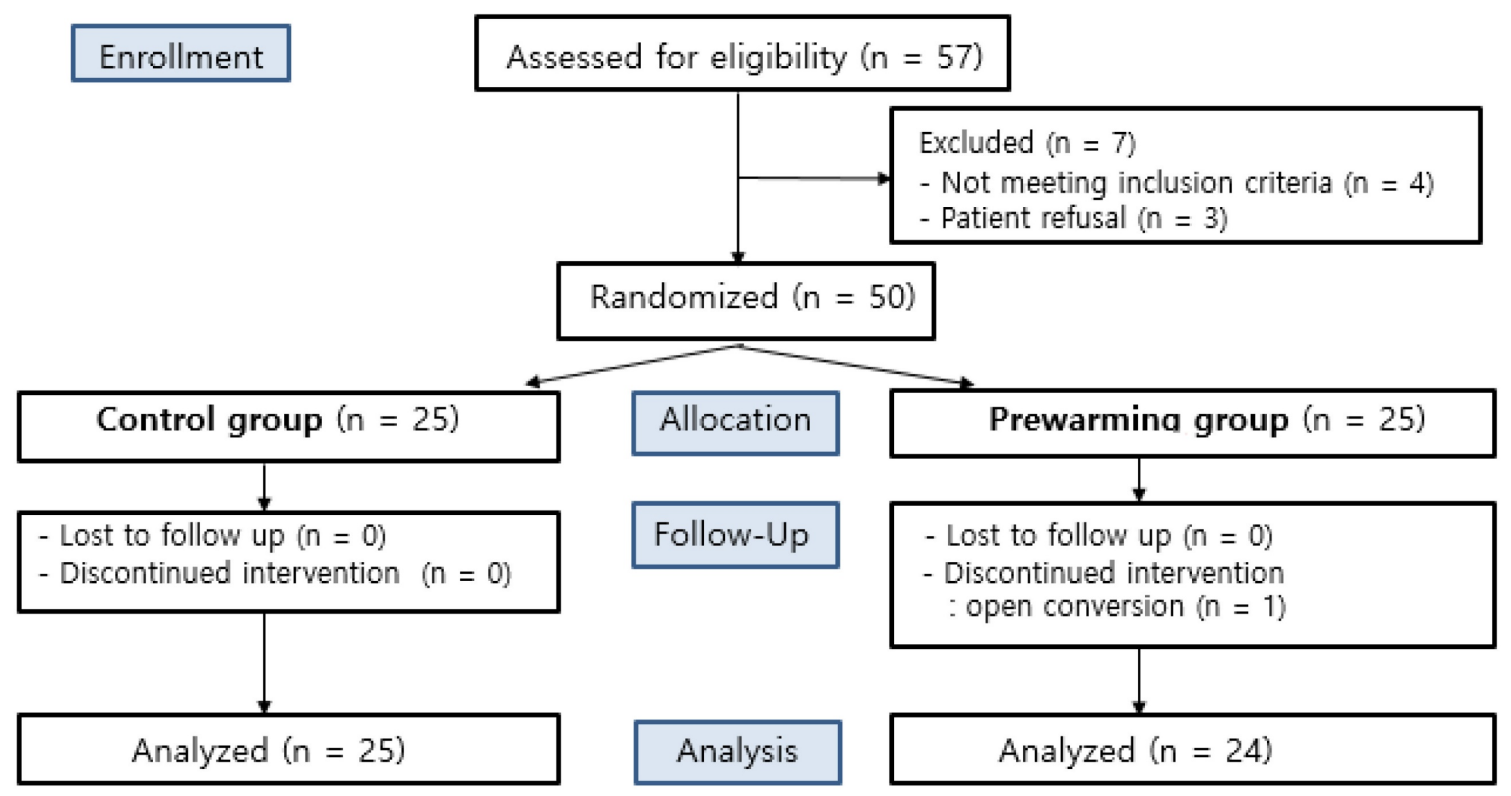
CONSORT flow gram.

A total of 57 patients undergoing TURB or TURP under general anesthesia were eligible to participate in this study. Seven patients were excluded according to the exclusion criteria, due to either a history of systemic disease or refusal to participate. The other 50 patients were randomly allocated to either the control group or the prewarming group. One participant of the prewarming group was discontinued due to their conversion to an open surgery (Fig. 1).

The demographic and perioperative data were not statistically significantly different between the groups except for gender (P = 0.047), weight (P = 0.015), and American Society of Anesthesiologists physical status (ASA) classification (P = 0.02) (Table 1).

**Figure 2:**
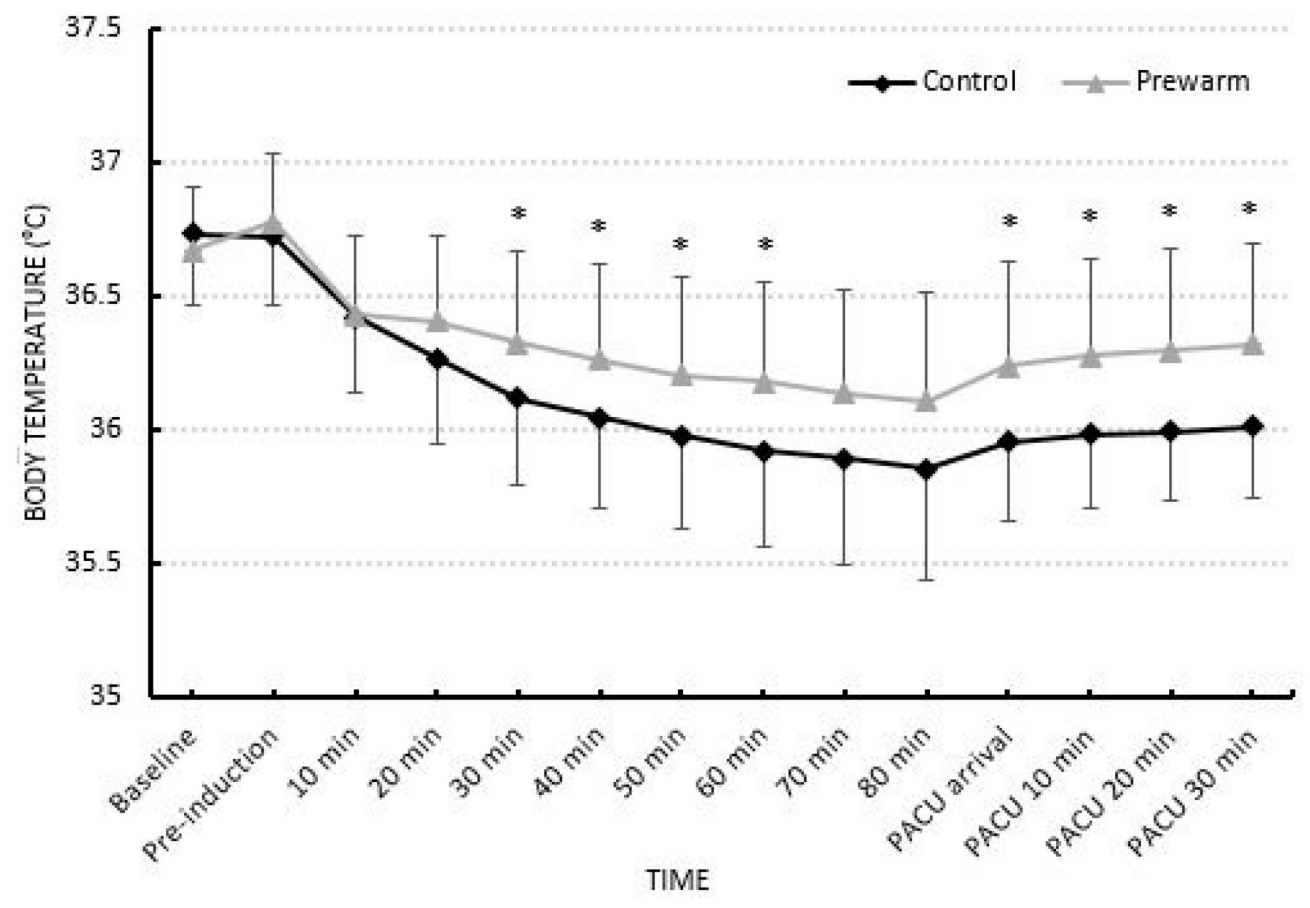
Comparison of change in core temperature. The bars are in mean ± standard deviation, * Difference between two groups with P < 0.05.

The incidence of hypothermia was higher in the control group (64%) than the prewarming group (29%) (P = 0.015). The prewarming group showed significantly less change in core temperature (0.55 ± 0.4°C, 0.93 ± 0.3°C, P = 0.0001), and core temperature drop rate than the control group (0.008 ± 0.004°C/min, 0.012 ± 0.004°C/min, P = 0.001) (Table 2). The core temperature of the two groups was statistically significantly different at 30, 40, 50, and 60 minutes after anesthesia induction and throughout the PACU stay (Fig. 2).

Of the postoperative outcomes, thermal comfort was statistically significantly different between the groups (P = 0.004) (Table 3). In the control group, there was one case of postoperative nausea and vomiting, one case of reoperation within a week, and two cases of postoperative delirium.

Comparative analysis of demographics and perioperative variables of normothermia patients and hypothermia patients was performed (Table 4). The incidence of reoperation and delirium was higher in hypothermia group but they were not statistically significant.

## Discussion

Ten minutes of prewarming combined with the administration of warmed intravenous fluid decreased the incidence and severity of intraoperative hypothermia and improved postoperative patient thermal comfort.

Previous studies evaluating the efficacy of 10 minutes of prewarming at preventing hypothermia reported inconsistent results 10-12, 15. Yoo et al. reported that 10 minutes of prewarming had no effect on the prevention of intraoperative hypothermia 12. In comparison, Lee et al. reported that 10 minutes of prewarming helped prevent hypothermia 10. Kawanishi et al. also reported that 10 minutes of prewarming was effective at maintaining normothermia 15. Kawanishi et al. reported a core temperature change from anesthesia induction (prewarming: 0.3 [0.3]°C, control: 0.6 [0.2]°C, P = 0.02) was less than in our study (prewarming: 0.55 ± 0.4°C, control: 0.93 ± 0.3°C, P = 0.0001) probably due to the fact that they analyzed patients from different populations undergoing different operations. The bladder irrigation utilized during the operation and the older group of patients in our study may have contributed to greater core temperature changes.

While patients are vulnerable to developing perioperative hypothermia during transurethral urologic surgeries, there is no standard prewarming method. To maximize the effect of quick prewarming without requiring additional expenses or interfering with the operation schedule, we administered warmed intravenous fluid. This combination showed positive results. While prewarming prevented distribution hypothermia, warmed intravenous fluid administration was an active warming method. A systematic review of eight trials comparing the effects of warmed and room temperature intravenous fluids showed that those receiving warmed intravenous fluid were half a degree warmer 60 minutes after anesthesia was induced than those receiving room temperature fluid 16. A continuous intravenous fluid warming device may deliver warmer fluid to patients than prewarmed fluid 17. However, the duration of the operation in this study was not long (< 1 hour), so prewarmed fluids were warm enough during the operation to help maintain normothermia. Warming is subject to a ceiling effect, so past a certain point, using more warming methods does not meaningfully affect core temperature 16. Warming patients in high temperature for too long can cause vasodilation or sweating and interfere with maintaining normothermia 7, 18. The present study combined warming methods that can be efficiently used for short operations, which is a strength of this study.

The results of our study showed a mean core temperature drop of 0.375°C. Although this statistically significant result is less than 0.5°C, which is the minimum therapeutic effect hypothesized from the sample size calculation, it was more than 0.2°C, which shows an important difference between an intervention and control group 9. Moreover, there was a statistically significant difference in the groups' thermal comfort, which is similar to the result found in a previous study on prewarming and thermal comfort 19. We suspect that the temperature difference between the groups was attenuated due to the universal application of intraoperative forced-air warming blankets to both groups. Also, patients were prewarmed in the preoperative holding area, so the prewarming effect may have decreased during patient delivery to the operating room.

This study had four major limitations. The first limitation was that we used a tympanic thermometer to measure the core temperature while patients were awake. While the gold standard in monitoring core temperature is measuring using a pulmonary artery catheter, using invasive measurements is impractical for low-risk patients and clinical guidelines indicate that the tympanic temperature is a reliable reflection of the core temperature. The measurement location is important for ensuring that infrared thermometer readings are reliable and we used the highest of three serially measured temperatures from an ipsilateral ear in all patients 4, 20. The second limitation was that patient body weight and ASA classification were significantly different between the groups. Obese patients better maintain body temperature with perioperative forced air warming 21. However, we assume that the weight difference between the groups was clinically insignificant as their body mass indices and administered intravenous fluid amounts were not statistically significantly different. An ASA classification over 1 is a risk factor for perioperative hypothermia 22, 23. Randomization seems to be the reason for the groups' statistically significantly different ASA classifications. Studies with better matching patient demographics would yield more objective results. For third, prewarming was performed in the preoperative holding area that there was warming interruption during transport to the operation room. However, we assume there is less likelihood of bias since all patients had surgery in the same operating room and were transported using the same path. For fourth limitation, our study population consisted of selected patients undergoing transurethral surgery under general anesthesia. Although irrigation fluids utilized during surgery augment hypothermia, anesthetics induced impairment of thermoregulation and vasodilation are the main contributor of perioperative hypothermia during general anesthesia 24. Thermal benefit of prewarming combined with warmed intravenous fluid is assumed to be also effective in other types of surgeries under general anesthesia.

## Conclusion

This study evaluated the efficacy of prewarming combined with warmed intravenous fluid administration at preventing intraoperative hypothermia in patients undergoing TURB or TURP under general anesthesia. The results show that this relatively simple intervention can effectively prevent intraoperative hypothermia and produce better patient thermal comfort in the PACU.

## Figures and Tables

**Table 1:** Demographic and perioperative data

Variables	Control group (n =25)	Prewarming group (n=24)	*P*
Age (years)	72.9 ± 9.4	67.9 ± 10.9	0.087
Sex (Male/Female)	19/6	23/1	0.047
Height (cm)	162.1 ± 8.2	166.3 ± 6.5	0.054
Weight (kg)	60.1 ± 9.1	67.8 ± 12.2	0.015
Body mass index (kg/m^2^)	22.8 ± 3.1	24.5 ± 3.5	0.076
Hypertension	14 (56)	10 (42)	0.316
Diabetes mellitus	9 (36)	9 (38)	0.913
ASA classification			0.020
II	16 (64)	22 (92)	
III	9 (36)	2 (8)	
Operation type TURB/TURP	20/5	21/3	0.478
Operation duration (min)	51.2 ± 31.5	45.5 ± 32.1	0.214
Anesthesia duration (min)	85.0 ± 31.8	75.8 ± 36.7	0.085
PACU time (min)	36.1 ± 8.6	36.9 ± 6.9	0.697
Crystalloid amount (mL)	652 ± 137.3	677.1 ± 130	0.515
Phenylephrine dose (μg)	45 ± 52	42 ± 67	0.544
Ephedrine dose (mg)	3.4 ± 4.7	1.9 ± 4.1	0.090
Irrigation fluid amount (mL)	10000 (4500-22500)	10000 (4250-15000)	0.857
Estimated blood loss (mL)	10 (5-40)	10 (5-30)	0.534
OR temperature (°C)	22.8 ± 0.9	22.5 ± 0.9	0.290
PACU temperature (°C)	22.9 (22.5-23.3)	22.8 (22-23)	0.256

Data are shown as mean ± standard deviation or median (interquartile range) or number (%). ASA: American Society of Anesthesiologists, TURB/TURP: transurethral resection of bladder/prostate, OR: operating room, PACU: postanesthesia care unit.

**Table 2:** Comparison of core temperature between two groups

	Control group (n = 25)	Prewarming group (n = 24)	95% CI	*P*
Hypothermia	16 (64)	7 (29)		0.015
Hypothermia severity (mild/moderate/severe)	16/0/0	7/0/0		0.015
Change in core temperature (°C)	0.93 ± 0.3	0.55 ± 0.4	0.19 to 0.56	0.0001
Core temperature drop rate (°C/min)	0.012 ± 0.004	0.008 ± 0.004	0.002 to 0.007	0.001

Data are shown as mean ± standard deviation, or number (%). Change in core temperature = T0-Tend, T0: tympanic temperature measured in the preoperative holding area, Tend: core temperature measured at the end of the operation, Core temperature drop rate = mean core temperature drop/anesthesia duration.

**Table 3:** Postoperative outcomes

Variables	Control (n =25)	Prewarming group (n=24)	*P*
shivering	2 (8)	0	0.157
thermal comfort			0.004
3 (moderately cold)	7	0	
4 (mildly cold)	4	1	
5 (neutral)	14	23	
PONV	1 (4)	0	0.322
reoperation	1 (4)	0	0.322
delirium	2 (8)	0	0.157

Data are shown as mean ± standard deviation or median (interquartile range) or number (%). PONV: postoperative nausea and vomiting, reoperation: hematoma removal within 1week, delirium: new onset delirium within postoperative 5 days.

**Table 4:** Subgroup analysis of demographics and perioperative data

Variables	Normothermia (n =26)	Hypothermia (n=23)	*P*
Age (years)	68.9 ± 11.0	72.2 ± 9.4	0.258
Sex (Male/Female)	22/4	20/3	0.815
Height (cm)	163.7 ± 8.2	164.6 ± 7.2	0.711
Weight (kg)	65.8 ± 12.4	61.6 ± 9.7	0.190
Body mass index (kg/m^2^)	24.4 ± 3.6	22.8 ± 2.9	0.086
ASA classification			0.052
II	23 (88)	15 (65)	
III	3 (12)	8 (35)	
Anesthesia duration (min)	75.5 ± 38.4	86.1 ± 28.7	0.284
Crystalloid amount (mL)	688.5 ± 141.6	636.9 ± 119.8	0.179
Irrigation fluid amount (mL)	15438.5 ± 17548.8	13460 ± 11726.9	0.650
OR temperature (°C)	22.7 ± 0.9	22.6 ± 0.9	0.614
PACU temperature (°C)	22.8 ± 0.7	22.8 ± 0.9	0.956
Reoperation	0 (0)	1 (4)	0.283
Delirium	0 (0)	2 (9)	0.125

Data are shown as mean ± standard deviation or number (%). ASA: American Society of Anesthesiologists, OR: operating room, PACU: postanesthesia care unit, reoperation: hematoma removal within 1week, delirium: new onset delirium within postoperative 5 days.

## Abbreviations

TURB: transurethral resection of the bladder
TURP: transurethral resection of the prostate
PACU: postanesthesia care unit
ASA classification: American Society of Anesthesiologists physical status classification
OR: operating room
PONV: postoperative nausea and vomiting
